# ATF6 as a Nodal Regulator of Proteostasis in the Heart

**DOI:** 10.3389/fphys.2020.00267

**Published:** 2020-04-08

**Authors:** Christopher C. Glembotski, Adrian Arrieta, Erik A. Blackwood, Winston T. Stauffer

**Affiliations:** Department of Biology, College of Sciences, San Diego State University Heart Institute, San Diego State University, San Diego, CA, United States

**Keywords:** ATF6, cardiac myocyte, proteostasis, ER stress, unfolded protein response

## Abstract

Proteostasis encompasses a homeostatic cellular network in all cells that maintains the integrity of the proteome, which is critical for optimal cellular function. The components of the proteostasis network include protein synthesis, folding, trafficking, and degradation. Cardiac myocytes have a specialized endoplasmic reticulum (ER) called the sarcoplasmic reticulum that is well known for its role in contractile calcium handling. However, less studied is the proteostasis network associated with the ER, which is of particular importance in cardiac myocytes because it ensures the integrity of proteins that are critical for cardiac contraction, e.g., ion channels, as well as proteins necessary for maintaining myocyte viability and interaction with other cell types, e.g., secreted hormones and growth factors. A major aspect of the ER proteostasis network is the ER unfolded protein response (UPR), which is initiated when misfolded proteins in the ER activate a group of three ER transmembrane proteins, one of which is the transcription factor, ATF6. Prior to studies in the heart, ATF6 had been shown in model cell lines to be primarily adaptive, exerting protective effects by inducing genes that encode ER proteins that fortify protein-folding in this organelle, thus establishing the canonical role for ATF6. Subsequent studies in isolated cardiac myocytes and in the myocardium, *in vivo*, have expanded roles for ATF6 beyond the canonical functions to include the induction of genes that encode proteins outside of the ER that do not have known functions that are obviously related to ER protein-folding. The identification of such non-canonical roles for ATF6, as well as findings that the gene programs induced by ATF6 differ depending on the stimulus, have piqued interest in further research on ATF6 as an adaptive effector in cardiac myocytes, underscoring the therapeutic potential of activating ATF6 in the heart. Moreover, discoveries of small molecule activators of ATF6 that adaptively affect the heart, as well as other organs, *in vivo*, have expanded the potential for development of ATF6-based therapeutics. This review focuses on the ATF6 arm of the ER UPR and its effects on the proteostasis network in the myocardium.

## Proteostasis and Proteotoxicity

**FIGURE 1:**
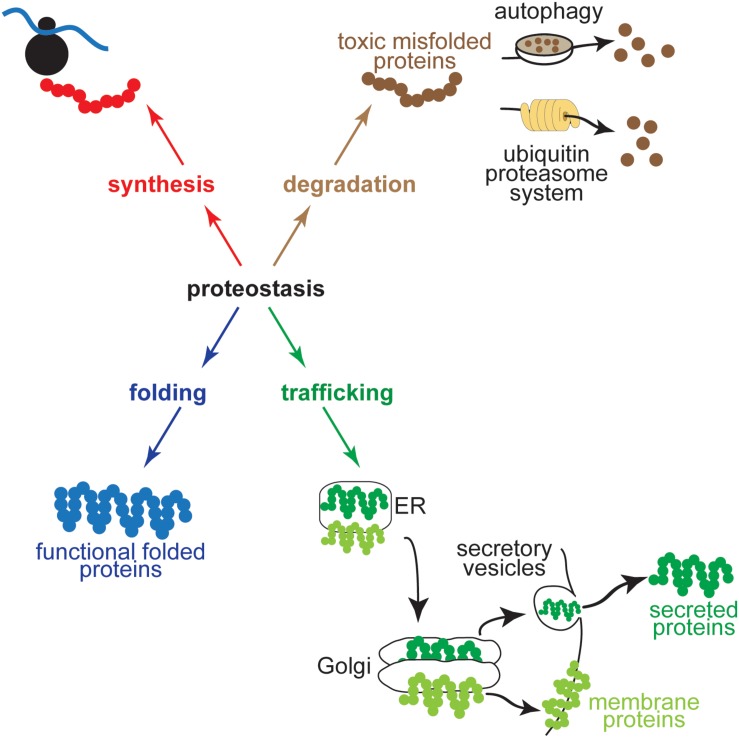
Proteostasis encompasses the cellular networks that contribute to protein synthesis, folding, trafficking, and degradation. Shown for protein synthesis **(red)** is a ribosome in the process of translation, for protein folding **(blue)** is a protein in its folded, functional conformation, for trafficking **(green)** are a secreted protein and a transmembrane protein moving through the ER secretory pathway to the Golgi before being routed to their final destinations in secretory vesicles or embedded in the plasma membrane, and for degradation **(brown)** is a toxic misfolded protein being degraded by an autophagosome by autophagy **(upper)** or by a proteasome via the ubiquitin proteasome system **(lower)**.

The integrity of the proteome in cardiac myocytes is critical for normal heart function. Proteome integrity in all eukaryotic cells is maintained by proteostasis, which encompasses the cellular networks that contribute to protein synthesis, folding, trafficking, and degradation (Balch et al., 2008; Sala et al., 2017; Figure 1). An imbalance amongst the components of these networks can lead to the accumulation of misfolded proteins and proteotoxicity or proteinopathy (Hightower, 1991; Douglas and Cyr, 2010), which in cardiac myocytes is associated with ischemic heart disease, as well as hypertrophic and dilated cardiomyopathies (McLendon and Robbins, 2015; Arrieta et al., 2018). At the least, the misfolding of proteins can impair their functions, but of potentially greater impact is that misfolded proteins can form toxic polypeptides, aggregates, and pre-amyloid oligomers inside and outside of cells that broadly affect cardiac myocyte function and viability, leading to heart failure (Wang and Robbins, 2006; McLendon and Robbins, 2011; Parry et al., 2015). In addition to heart disease, impaired proteostasis has been linked to numerous other pathologies including atherosclerosis, diabetes, fatty liver disease, and neurodegenerative diseases (Wang and Kaufman, 2012, 2014, 2016; Rivas et al., 2015; Hetz and Saxena, 2017; Valenzuela et al., 2018); moreover, impaired proteostasis can occur as a function of the aging process (Kikis et al., 2010; Lakatta, 2015; Hipp et al., 2019).

Since disease- and age-related protein misfolding and proteotoxicity has been found in many organs, numerous studies have focused on discovering components of the proteostasis network in hopes of identifying potential targets for therapies to minimize the untoward effects of proteotoxicity on organ function. These studies have revealed that mechanisms responsible for the surveillance of the structural integrity of nascent and mature proteins, as well as the processes that determine the fate of terminally misfolded proteins, reside in many cellular locations (Labbadia and Morimoto, 2015). One fate of terminally misfolded proteins is degradation by the ubiquitin proteasome system (UPS), the components of which are localized to specific regions of cells (Pohl and Dikic, 2019). For example, proteasomes have been found on cardiac myocyte contractile elements, primarily at the Z-line of sarcomeres and in the cytosol and nucleus, as well as decorating the surface of many organelles, such as the ER, mitochondria, and lysosomes (Brooks et al., 2000; Wojcik and DeMartino, 2003; Glembotski, 2012a; Bard et al., 2018). However, toxic proteins must still be degraded, even if they are generated in cellular regions that do not have proteasomes. For example, terminally misfolded proteins can be degraded in a proteasome-independent manner by autophagy (Chen et al., 2019). Organelle- and subcellular-specific forms of autophagy, such as mitophagy (Gustafsson and Dorn, 2019) and ER-autophagy, or reticulophagy (Wilkinson, 2019), also degrade terminally misfolded proteins and in so doing, they contribute to maintaining proteome integrity.

Most proteins fold co-translationally and in some cases, folding into the final active configuration is a molecular trial-and-error process (Choi et al., 2013). In fact, it is thought that protein-folding process results in as much as 30% of proteins never reaching their active folded configurations; such proteins are degraded either during or soon after translation (Schubert et al., 2000). This suggests that the elements of the proteostasis network that maintain proteome integrity must be physically located near nodal points of protein synthesis. Since secreted and membrane proteins, which account for as much as 40% of proteins made in eukaryotic cells (Fregno and Molinari, 2019), are synthesized at the endoplasmic reticulum (ER), the ER is a natural node for the cellular proteostasis network. In cardiac myocytes, the ER includes an extensive sarcoplasmic reticulum involved in contractile calcium handling (Bers, 2002a,b). However, while it has been less studied than calcium handling, the ER in cardiac myocytes is also important for the synthesis of many membrane and secreted proteins that are important for viability and contractile function, including hormones, growth factors and stem cell homing factors, as well as ion channels and many other proteins that are critical for excitation-contraction coupling (Glembotski, 2012b).

## ER Stress and the Unfolded Protein Response

Soluble ER proteins, which include secreted and ER-resident proteins, and membrane proteins are made on ER-bound ribosomes, where they are co-translationally translocated through an ER membrane channel, the translocon, across the ER membrane into the lumen of the ER, or they are embedded in the ER membrane (Lingappa and Blobel, 1980; Kelly, 1985; Nicchitta, 2002; Egea et al., 2005; Viotti, 2016; Glembotski, 2017). This is followed by continued folding of the nascent proteins, as well as post-translational modifications, such as glycosylation, phosphorylation, disulfide bond formation, and proteolytic processing, most of which also affect the folding process and take place *en route* to their final destinations (Braakman and Bulleid, 2011; Steiner, 2011). Conditions that alter the environment in the ER in ways that impair any of these processes can cause ER stress, which can lead to the accumulation of potentially proteotoxic misfolded proteins in the ER lumen or membrane (Paschen and Doutheil, 1999; Welihinda et al., 1999; Hampton, 2000; Urano et al., 2000; Berridge, 2002; Rutkowski and Kaufman, 2004). Conditions that place higher demands on the ER protein-synthesis, -trafficking and -routing machinery, such as high levels of protein synthesis at the ER, can also lead to ER stress (Oakes, 2017; Su and Dai, 2017). For example, β-cells of the pancreas make so much insulin, which is synthesized and trafficking by the ER/Golgi secretory pathway that they are continually under ER stress (Iwawaki et al., 2004; Eizirik and Cnop, 2010; Hodish et al., 2011).

**FIGURE 2:**
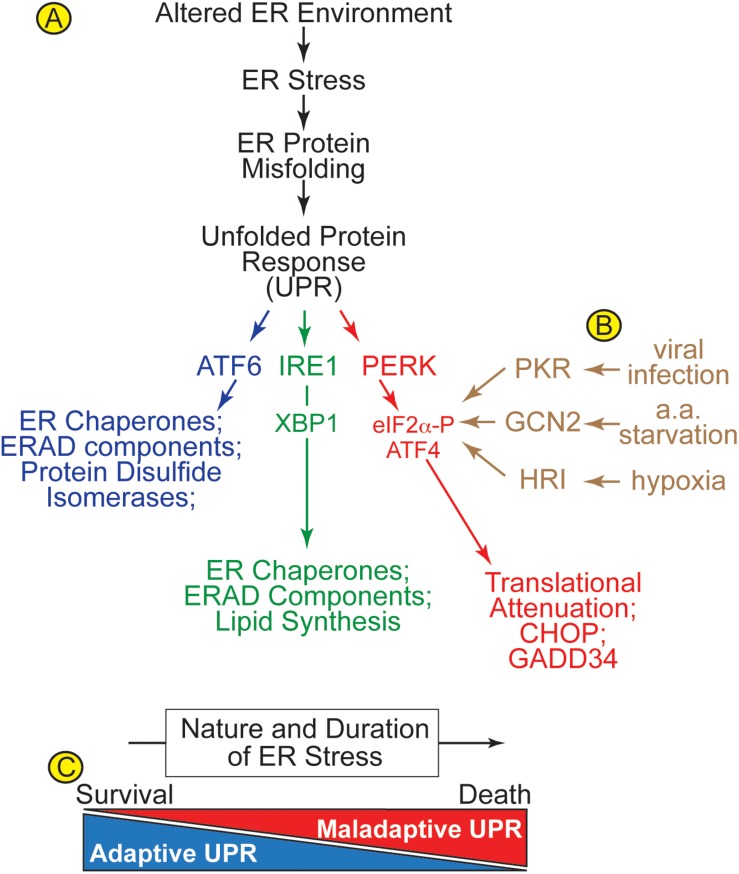
**(A)** Alterations in the ER environment cause ER stress and ER protein misfolding, which activates three arms of the unfolded protein response (UPR) via three ER transmembrane proteins, ATF6, IRE1, and PERK. **(B)** The integrated stress response includes ER stress, viral infection, amino acid starvation, and hypoxia, which activate four different kinases, PKR-like ER kinase (PERK), protein kinase double stranded RNA-dependent (PKR), general control non-derepressible-2 (GCN2), and heme-regulated inhibitor (HRI), respectively, all of which converge on the phosphorylation of eIF2α on Ser-51. **(C)** Acute ER stress activates survival-oriented adaptive aspects of the UPR, while chronic ER stress leads to death-oriented maladaptive aspects of the UPR. The balance between adaptive and maladaptive UPR pathways is determined by the nature and duration of the ER stress.

When the ER environment is altered in ways that cause ER stress, ER protein misfolding activates the ER unfolded protein response (UPR) (Figure 2A). There are three main branches of the UPR that are regulated by ER transmembrane protein sensors of ER stress; ATF6α (activation of transcription factor 6, called ATF6 from here on), PERK [protein kinase R (PKR)-like kinase, and IRE1 (inositol requiring enzyme 1)] (Glembotski, 2007; Hetz and Glimcher, 2011; Walter and Ron, 2011). ATF6 is a transcription factor (Wang et al., 2000; Glembotski, 2014). IRE1 is a nuclease that splices the XBP1 mRNA so it encodes an active transcription factor called XBP1 spliced (XBP1s) (Urano et al., 2000). PERK is a kinase that phosphorylates the translation initiation factor, eIF2α on Ser-51, which causes global translational arrest, but allows for the continued translation of a select subset of mRNAs that encode proteins that are necessary for the adaptive UPR (Schroder and Kaufman, 2006). Many of the genes induced by ATF6 and XBP1s, as well as other events that lie down stream of PERK, are initially oriented toward restoring ER protein folding. Such genes that are upregulated during the initial phases of UPR activation encode ER chaperones, protein disulfide isomerases (PDIs), and proteins involved in ER associated protein degradation (ERAD). ERAD is a specialized form of protein degradation that involves the retrotranslocation of misfolded proteins out of the ER lumen or membrane, followed by their ubiquitylation by ER-bound E3-ubiquitin ligases on the cytosolic face of the ER, which marks them for degradation by proteasomes, also located on the cytosolic face of the ER (Hampton, 2002; McCracken and Brodsky, 2003; Ahner and Brodsky, 2004; Meusser et al., 2005; Kuhnle et al., 2019).

Many early studies of the UPR used chemical inducers of ER stress, such as thapsigargin and tunicamycin (D’Amico et al., 1992; Wong et al., 1993), which are more robust activators of the UPR than physiological or pathological conditions that activate the UPR. These early studies showed that many of the effects of the different branches of the UPR overlap, i.e., induction of ER chaperones and ERAD components by ATF6 and IRE1 branches, while others did not, e.g., induction of ATF4 and CHOP by PERK activation (Figure 2A). However, as the field matured it was found that the effects of each branch of the UPR can be quite different when observed under more subtle stress conditions, including those that occur during pathology. For example, amongst the three branches of the UPR, the IRE1/XBP1 arm is a specific inducer of genes involved in protein O-GlcNAcylation in the ischemic heart (Wang et al., 2014), while ATF6 is an inducer of certain antioxidant genes during myocardial ischemia/reperfusion (I/R) (Jin et al., 2017). Studies such as these provide evidence that the downstream effects of the three branches of the UPR probably overlap less than originally observed when thapsigargin and tunicamycin were used to elicit ER stress. Contributing further to this complexity is the finding that ATF6 can induce XBP1 (Lee et al., 2002), and ATF6 can induce itself (Misra et al., 2013), underscoring the not-well-understood autoregulation that exists amongst the UPR branches (Brewer, 2014). Further complicating matters is the fact that, in addition to PERK, there are at least three other kinases known to phosphorylate eIF2α as part of the integrated stress response, one component of which is ER stress (Figure 2B; Donnelly et al., 2013). Moreover, the temporal dynamics with which the three branches of the UPR are activated differs, depending on the cell type, as well as the nature, strength, and duration of the stress (Marton et al., 2017; Sharma et al., 2019). These temporal dynamics appear to dictate whether the UPR is adaptive or maladaptive (Figure 2C). The adaptive UPR is the first to be activated, and its major role is to restore ER proteostasis, improve protein folding and avoid proteotoxicity. However, if the adaptive UPR does not sufficiently fortify the ER protein-folding machinery, then continued ER stress is associated with a conversion of UPR signaling from adaptive to maladaptive, as the downstream events that are regulated by the UPR shift from being survival oriented to cell death oriented (Sano and Reed, 2013).

## ATF6 is an Adaptive Responder to ER Stress

### Mechanism of ATF6 Activation During ER Stress

The focus of this review is the ATF6 arm of the UPR, which has major adaptive effects in numerous cell types (Wu et al., 2007; Yamamoto et al., 2007; Wang et al., 2018; Glembotski et al., 2019). The activation of ATF6 as a transcription factor takes place very soon after the onset of ER stress. At this time ATF6 regulates a gene program that fosters adaptive UPR responses, in part because so many ER-resident protein-folding proteins, such as chaperones, are induced at this early time by ATF6 (Mao et al., 2006; Martindale et al., 2006). Moreover, several studies have examined the effects of ATF6 in the heart in mice, *in vivo*, and have revealed the adaptive effects of ATF6 during physiological and pathological conditions (Martindale et al., 2006; Jin et al., 2017; Yu et al., 2017; Blackwood et al., 2019a, b).

**FIGURE 3:**
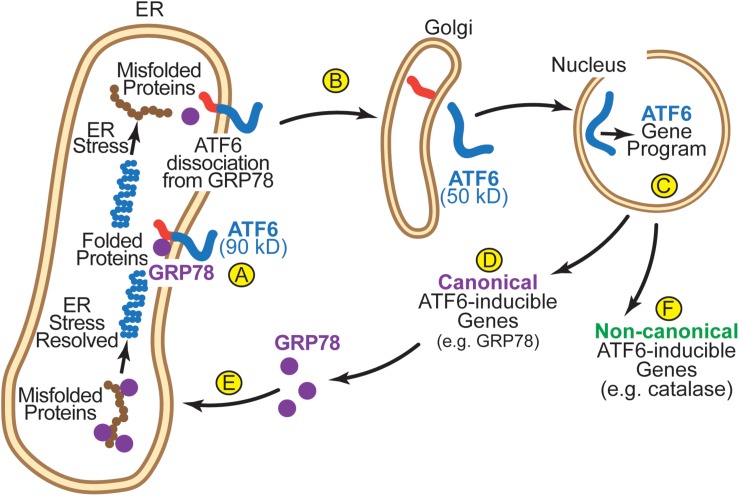
**(A)** In its inactivated state, ATF6 is a 90 kD ER transmembrane protein that is anchored in the membrane by GRP78. **(B)** Upon ER stress, GRP78 dissociates from the ER luminal domain of ATF6, which allows the 90 kD form of ATF6 to translocate to the Golgi, where is it cleaved by S1P and S2P to liberate the N-terminal approximately 400 amino acids (50 kD) of ATF6 from the ER membrane. **(C)** The clipped form of ATF6 has a nuclear localization sequence, which facilitates its movement to the nucleus where it binds to specific regulatory elements in ATF6-responsive genes, such as ER stress response elements (ERSEs), and induces the ATF6 gene program. **(D)** The canonical ATF6 gene program comprises genes that encode proteins that localize to the ER, such as the chaperone, GRP78 **(E)**, where they fortify ER protein folding. **(F)** The non-canonical ATF6 gene program comprises genes that encode proteins not typically categorized as ER stress-response proteins, such as catalase, which localize to regions of the cell outside the ER.

A number of studies have elucidated the mechanism of ATF6 activation. In the absence of ER stress, inactive ATF6 is a 90 kD ER transmembrane protein (Haze et al., 1999) (Figure 3A). Upon ER stress, ATF6 senses the accumulation of misfolded proteins in the ER. In part, this sensing mechanism involves the ER chaperone, GRP78. In the absence of ER stress, GRP78 binds to the ER luminal domain of ATF6 and, by virtue of the ER-retention sequence at the C-terminus of GRP78 it anchors ATF6 in the ER, as ATF6 has no known ER-retention sequence (Chen et al., 2002; Shen et al., 2005). Upon ER stress GRP78 dissociates from, and thus, releases ATF6, allowing it to relocate to the next destination in the secretory pathway, the Golgi (Figure 3B; Shen et al., 2002). In the Golgi, ATF6 is cleaved by S1P and S2P proteases by regulated intramembrane proteolysis (Ye et al., 2000). This cleavage liberates the N-terminal 50 kD cytosolic portion of ATF6, which has a nuclear localization sequence, facilitating its movement to the nucleus, where it acts as a transcription factor to induce genes that encode proteins that fortify the ER protein-folding environment (Figure 3C; Haze et al., 1999). In the heart, ATF6 has been shown to induce many genes that are known to serve roles in the adaptive UPR (Belmont et al., 2008; Blackwood et al., 2019b). These canonical ATF6-inducible genes encode proteins, most of which are ER-resident chaperones (e.g., GRP78), PDIs, and ERAD components (Figure 3D) that localize to the ER where they contribute to restoring ER protein folding (Figure 3E). However, it was found in the heart that ATF6 also induces non-canonical adaptive genes that were not previously known to be ER stress-response genes, many of which encode proteins that do not even reside in the ER (Figure 3F; Jin et al., 2017).

### ATF6 Is Rapidly Degraded When Activated

One of the most fascinating findings regarding ATF6 was the discovery in model cell lines that the active form of ATF6 is rapidly degraded (Thuerauf et al., 2002). In fact, the degradation is so rapid that it is difficult to find the cleaved form of ATF6 by immunoblotting unless proteasome inhibitors are used. The rapid degradation of ATF6 suggests that its activity is designed to be transient; although the reasons for this are not yet known, it is apparent that this transient activation leads to transient induction of ATF6 responsive genes, and that this temporal sequence must be an important determinant of the effects of these genes. In addition to ATF6, there are a number of other transcription factors that are rapidly degraded, once they are activated (Geng et al., 2012). Thus, while ATF6 is not unique in this regard, functional mapping of the domains of ATF6 have led to novel findings regarding the mechanism by which ATF6 transcriptional activity and degradation are regulated. These mapping studies, done in cell lines, demonstrated that within the N-terminal transcriptional activation domain of ATF6 is a stretch of 8 amino acids with a sequence that is very similar to a sequence of amino acids found in the rapidly degraded viral transcription factor, VP16 (Thuerauf et al., 2002). In VP16, this eight amino acid region is responsible for its potent transcriptional activity, as well as its rapid degradation (Tanaka, 1996). Mutation analyses showed that as in VP16, this region of ATF6 is responsible for its transcriptional activity and its rapid degradation (Thuerauf et al., 2002). In fact, detailed functional mapping of ATF6 showed that the transcriptional activation domain also serves as the signal for rapid degradation. Additional studies demonstrated that ATF6 was rapidly degraded only when it was actively engaged in transcriptional activation, and that any mutations introduced into ATF6 that decreased it transcriptional activity coordinately increased its half-life (Thuerauf et al., 2007). Finally, a different form of ATF6, called ATF6β, which is also activated during ER stress, does not have the VN8 region but is similar to ATF6 in many other regions. It was further shown that it was because ATF6β does not have the VN8, it has very low transcription factor activity and it is slowly degraded, thus it has molecular characteristics that oppose those of ATF6a. In fact, since ATF6 functions as a dimer, and since it can dimerize with ATF6β, the relative amounts of these two forms of ATF6 can dictate the composition of ATF6 dimers in ways that determine the strength with which the ATF6 gene program is induced (Thuerauf et al., 2007). For example, in that study it was shown that the transcription factor activity and the stability of dimers decreased coordinately in the following order: ATF6-ATF6 > ATF6-ATF6β > ATF6β-ATF6β. Thus, it is possible that if ATF6β is activated by ER stress at a slightly later time than ATF6 it may contribute to decreasing the transcriptional induction effects of ATF6, thus ensuring the transient and, thus, adaptive nature of ATF6-mediated gene induction.

### Transgenic Mice Reveal Adaptive Roles for ATF6 *in vivo*

While Mice Reveal Adaptive Roles for ATF6 *in vivo* the precise reasons for the transient nature of ATF6 activation are yet to be determined, and while the importance of the functional and physical interactions between ATF6 and ATF6β are not completely understood, it is apparent that the relatively brief time of ATF6 activation after ER stress must be important for its adaptive properties. Based on this premise, the ability to carefully regulate the extent of ATF6 activation was a consideration when ATF6 transgenic mice were prepared (Martindale et al., 2006). Accordingly, in those studies the transgenic mice were designed so they express the active form of ATF6 fused to the mutant mouse estrogen receptor (MER), which can bind tamoxifen (Zhang et al., 1996). Based on other studies with MER fusion protein expression in mice, it was reasoned that in the absence of tamoxifen, ATF6-MER would assume a conformation that blocks its transcriptional activity. However, when tamoxifen binds to ATF6-MER, the conformation would change to an active form of ATF6. Accordingly, to study the function of activated ATF6 in cardiac myocytes, *in vivo*, the construct used to generate the mice was prepared so that ATF6-MER expression would be under the control of the αMHC gene, which specifies cardiac myocyte-specific expression (Martindale et al., 2006). Indeed, tamoxifen administration for short periods of time led to the transcriptional induction of known ATF6 gene targets through regulatory regions in the genes called ER stress response elements, or ERSEs. Interestingly, ATF6-MER was not only a robust transcription factor, but it was rapidly degraded; however, both of these properties were dependent upon tamoxifen administration. This demonstrated that, in addition to cultured model cell lines, the degraded-when-active property of ATF6 could also be observed in cardiac myocytes, *in vivo*. Accordingly, this ATF6-transgenic (TG) mouse model allowed the precise temporal regulation of ATF6 activation in the heart in the absence of ER stress, so the function of only the ATF6 arm of the UPR in cardiac myocytes could be determined. Moreover, this mouse model facilitated the identification of genes that are regulated by ATF6 in the mouse heart, *in vivo*.

## Roles for ATF6 in the Heart

### ATF6 Is Activated by Ischemia/Reperfusion and Is Protective

**FIGURE 4:**
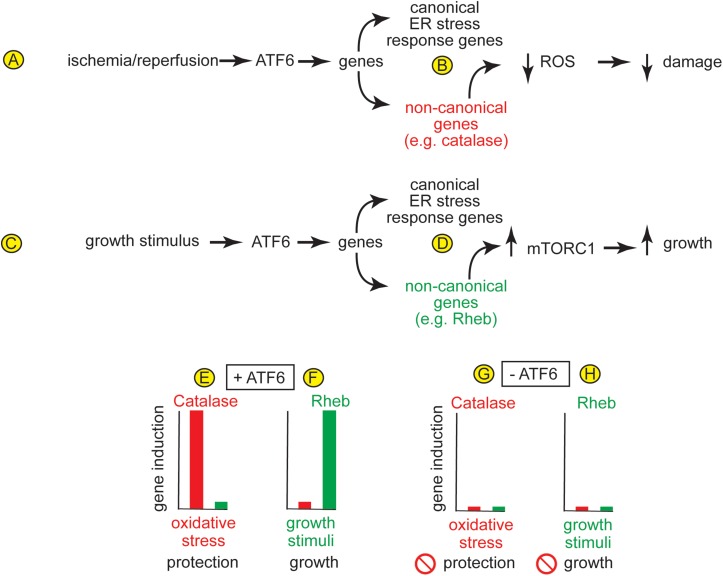
**(A)** In the heart, ischemia/reperfusion activates ATF6, which **(B)** induces canonical ER stress-response genes, as well as non-canonical genes, such as the peroxisomal antioxidant, catalase, which resides in peroxisomes and protects from the ROS that are generated during I/R. **(C)** In the heart, growth stimuli activate ATF6, which **(D)** induces canonical ER stress-response genes, as well as non-canonical genes, such as the small GTP-binding protein, Rheb, which is an activator of the growth-promoting kinase complex, mTORC1. **(E,F)** In the heart, different stimuli induce different ATF6-dependent genes. For example, **(E)** oxidative stress induces catalase but not rheb, and catalase protects the heart from damage, while **(F)** growth stimuli induce rheb but not catalase, which is required for physiological and pathological hypertrophic growth of the heart. **(G,H)** The absence of induction of any of these genes when ATF6 is deleted from cardiac myocytes supports the view that gene induction by both oxidative stress and growth stimuli is dependent upon ATF6.

Soon after the ATF6-MER transgenic mice were developed they were used to determine the role for activated ATF6 in cardiac myocytes, *in vivo*. It was found that activated ATF6 induced canonical ATF6-dependent ER stress response genes, such as ER chaperones, and conferred protection from I/R damage in *ex vivo* isolated perfused heart preparations and maintained contractile function (Martindale et al., 2006). This was the first report that activated ATF6 could be protective in any tissue, *in vivo*. These findings were coupled with other studies showing that in wild type mice, I/R could activate ATF6 (Blackwood et al., 2019a), leading to the hypothesis that when I/R activates ATF6, the genes induced by ATF6 contribute to protection against I/R damage and, thus play a role in maintaining cardiac function (Figure 4A). A subsequent study that also indicated that ATF6 reduces damage in the heart, examined the effects of ATF6 inhibition using either a chemical inhibitor of ATF6 or transgenic overexpression of dominant negative ATF6 in a mouse model of myocardial infarction (Toko et al., 2010). That study showed that inhibiting ATF6 increased the damage after MI. Another study examined roles for ATF6 outside the heart, where ATF6-MER was expressed specifically in mouse forebrain neurons *in vivo*. In that study, activated ATF6 protected neurons from damage a mouse model of ischemic stroke (Yu et al., 2017). These findings stimulated the search for the mechanism of protection from I/R damage. Since I/R damage is caused mainly by the generation of damaging reactive oxygen species (ROS) by mitochondria during reperfusion, it was not obvious how the canonical roles for ATF6, such as the induction of ER chaperones, like GRP78, could contribute to protection. Accordingly, transcriptomics approaches were undertaken in hopes of finding genes that might contribute to the adaptive effects of ATF6, *in vivo*.

## Mechanisms of the Adaptive Effects of ATF6 in the Heart

### Discovery of Non-canonical Roles for ATF6 in the Heart

Initial microarray studies to identify the genes induced by ATF6 in the hearts of ATF6-MER mice provided a wealth of information, the most provocative being that there were numerous genes induced by ATF6 that encoded proteins that were not previously known to be ATF6- or ER stress-inducible, and did not code for proteins that reside in the ER. These non-canonical ATF6-inducible genes provided the first clue that in the mouse myocardium, the function of ATF6 was much broader than the canonical ER stress response (Belmont et al., 2008). For example, in that study it was shown that ATF6 might affect myocardial growth by inducing the NFAT regulator, RCAN. Another study used a micro-RNA array analysis to define the microRNAs regulated by ATF6, and in the process demonstrated that one ATF6-downregulated microRNA was specific for the ER luminal calcium-binding protein, calreticulin, which implicated ATF6 as a regulator of the expression of calcium-handling proteins in the heart (Belmont et al., 2012).

### ATF6 Induces Antioxidant Genes in the Heart

As a complement to the ATF6 gain-of-function approach afforded by the ATF6-MER mice, ATF6 gene deletion was used to determine roles for endogenous ATF6 in mouse hearts under physiological and pathological conditions. It was found that ATF6 knockout mice (ATF6 KO) exhibited greater damage in mouse models of *ex vivo* and *in vivo* myocardial I/R, which was also consistent with the hypothesis that in the heart, I/R-mediated ATF6 activation led to the induction of genes that could protect against I/R damage (Jin et al., 2017). In that same study, transcriptome analyses provided a surprising result, that in cardiac myocytes, in addition to canonical ATF6-inducible genes, like GRP78, ATF6 induced numerous antioxidant genes, one of which encodes the potent antioxidant enzyme, catalase (Figure 4B). This study went on to show that catalase is a previously unidentified ER stress response gene, and that it is induced in the heart in an ATF6-dependent manner during I/R. Supporting this assertion was the finding that the addition of exogenous catalase restores protection against I/R damage to the hearts of mice in which ATF6 and been deleted, demonstrating that it is a least partly by virtue of inducing catalase induction that ATF6 mitigates the generation of ROS and reduces I/R damage in the mouse heart (Jin et al., 2017). It is interesting to note that catalase is not an ER-resident protein, but is instead normally expressed in peroxisomes (Poole et al., 1969). Accordingly, the catalase in peroxisomes serves to neutralize some of the ROS generated in mitochondria during I/R in the heart.

### ATF6 Is Required for Hypertrophic Growth of the Heart

Taking the ATF6 gene deletion studies a step further were studies in which ATF6 was conditionally deleted specifically from cardiac myocytes (ATF6 cKO) (Blackwood et al., 2019b). Compared to wild type mice, myocardial damage was exacerbated in ATF6 cKO mice subjected to I/R *in vivo* (Blackwood et al., 2019b). These findings corroborated the studies with ATF6 KO mice and further supported that ATF6-induced genes that protected from I/R damage (Jin et al., 2017). However, another surprise finding was that cardiac specific deletion of ATF6 decreased hypertrophic growth of the heart *in vivo* upon short times of pressure overload-induced pathological hypertrophy and decreased cardiac function (Blackwood et al., 2019b). In fact, physiological cardiac hypertrophy in mice subjected to free-wheel exercise was lower in the hearts of ATF6 cKO mice compared to control mice, supporting the hypothesis that growth stimuli activate ATF6-dependent genes that are required for cardiac myocyte growth (Figure 4C). In that study, transcript profiling revealed that, in addition to canonical ATF6-inducible genes, like GRP78, some of the non-canonical genes induced by ATF6 in mouse hearts were growth regulators, one of which was the small GTP-binding protein, Rheb (Figure 4D). Rheb had previously been shown to be a required activator of the growth-promoting kinase, mTORC1 in neurons (Yamagata et al., 1994). In the heart, it was shown that ATF6 was required to induce Rheb and, thus, mTORC1-dependent growth in acute models of pressure-overload induced pathological hypertrophy and in longer-term freewheel exercise-induced physiological hypertrophy (Blackwood et al., 2019b). In that study, further mechanistic examination showed that it is the increase in protein synthesis during hypertrophic growth of the heart that increases the protein-folding demand in cardiac myocytes, leading to activation of ATF6 and the induction of Rheb and activation of mTORC1. Similar results were found in a more recent publication, where it was shown that deletion of ATF6α or ATF6β resulted in a reduction of pathological cardiac hypertrophy at early times after pressure overload (Correll et al., 2019). In that report it was concluded that deletion of either ATF6α or ATF6β had a similar impact on hypertrophy, suggesting some redundancy in their functions.

## Different ATF6 Activators Induce Different ATF6-Dependent Gene Programs

While activation of ATF6 in the ATF6-MER TG mice was a useful model system for identifying numerous genes that could be induced specifically by the ATF6 arm of the UPR in the heart, it was of interest to determine what ATF6-dependent genes were induced under physiological and pathophysiological conditions that activate endogenous ATF6. Such studies led to one of the most remarkable discoveries, that different activators of ATF6 could induce different ATF6-dependent gene programs. For example, it was found that in mice treatments that cause oxidative stress, such as I/R, activate ATF6, which induces the antioxidant, catalase, but not the growth-promoter, Rheb (Figure 4E). In contrast, treatments that stimulate growth, such as pressure overload or exercise, which also activate ATF6, induce Rheb but not catalase (Figure 4F). Importantly, ATF6 gene deletion showed that each of these stimulus-specific gene programs is ATF6-dependent (Figures 4G,H; Blackwood et al., 2019b). Further promoter analysis of these genes demonstrated that ATF6 bound specifically to ERSEs in the catalase and rheb gene promoters, but this binding occurred only when cells were subjected to oxidative stress or growth stimuli, respectively. These studies suggest that the ATF6 gene program is tailored to suit the needs of cells, which differ, depending on the form of stress. Further support of this is a study in yeast and mammalian cell lines showing that ATF6 can be activated by specific sphingolipids, such as dihydrosphingosine (DHS) and dihydroceramide (DHC), and this occurs in the absence of ER protein misfolding (Tam et al., 2018). It seems possible that different stress conditions that all activate ATF6 may differentially activate other, as yet unidentified components of ER stress-inducible transcriptional programs, the latter of which may be responsible for imparting stress-specific gene regulation to ATF6.

## ATF6-Based Small Molecule Therapies for Heart Disease

In more recent studies a chemical biology approach has been taken in an effort to capitalize on the beneficial effects of ATF6 activation for the development of potential therapeutics for heart disease. These studies began with the screening of a small molecule library of over 600,000 different compounds for those that would activate ATF6 in 293 cells (Plate et al., 2016). This study used a highly rigorous multiplex screening approach in hopes of finding compounds that would activate only the ATF6 arm of the UPR, without activating other signaling pathways and without cause cell death. While several compounds were initially identified, one of them, compound 147, was the most selective for ATF6 activation in 293 cells, as well as isolated cardiac myocytes. In subsequent studies it was shown that compound 147 could be administered to mice without any untoward effects in the absence of pathology; but in mice subjected to *in vivo* myocardial I/R, 147 administration decreased cardiac damage and improved contractile function (Blackwood et al., 2019a). In that study, it was shown that compound 147 also decreased damage and improved motor function in a mouse model of cerebral I/R and decreased kidney damage in a mouse model of renal I/R while improving glomerular filtration. A subsequent study of the mechanism of action of compound 147 showed that this compound itself is not the active species, but it is converted to the active compound by a cytochrome P450 enzyme that is specifically expressed in the ER (Paxman et al., 2018), which probably accounts for the high selectivity of compound 147 as an activator of ATF6. The results obtained so far with compound 147 suggest that it may be a good candidate for consideration as a treatment for ischemic disease and other pathologies involving protein misfolding, including cardiomyopathy and heart failure. Accordingly, next steps in moving compound 147 toward clinical application would involve animal studies of the pharmacological properties of compound 147, including pharmacokinetics and toxicology, as well as examining the effects of 147 in large animal models of disease, such as pig models.

## Conclusion and Future Directions

Like many signaling pathways, the UPR exhibits both adaptive and maladaptive activities depending upon the nature and duration of the conditions that activate the UPR. While it was important to discover the conditions leading to either the adaptive and maladaptive UPR, this dual nature of the UPR makes it difficult to design UPR-based therapeutics. The ATF6 arm of the UPR provides fertile ground for developing adaptive therapeutics because it is mostly known for its adaptive effects. However, even ATF6 probably has both adaptive and maladaptive properties, which may be why it exhibits a “degraded-when-active” property. Thus, it is reasonable to posit that a useful ATF6-based therapy should exhibit a rapid onset, as well as a relatively transient action, thus mimicking, to some extent, the way the adaptive effects of ATF6 are activated during ER stress. The development of gene therapy approaches to increase activated ATF6, while useful in experimental animal systems (Jin et al., 2017), may not represent the best therapeutic approach because of the relatively long-term effects of such therapies. However, the ability of a small molecule, like compound 147, to activate endogenous ATF6 in a relatively transient manner mimics the adaptive effects of ATF6 *in vivo* and as such, may represent a worthwhile direction for future therapeutic development (Blackwood et al., 2019a). Indeed, the lack of untoward effects of compound 147, coupled with its relatively brief action *in vivo* set the stage for a bright future for such an approach. Moreover, the search for better ATF6 activators, such as those that might capitalize on the stimulus-specific activation properties of ATF6 and induce the ATF6 gene program only during I/R, or pressure overload might be sought. Also, selective activators of the other arms of the UPR will likely result in the discovery of lead compounds, like 147, that can be used in therapeutic approaches aimed at capitalizing on the adaptive effects of the UPR in all tissue and cell types.

## Author Contributions

All of the authors participated in all aspects of preparing this manuscript.

## Conflict of Interest

The authors declare that the research was conducted in the absence of any commercial or financial relationships that could be construed as a potential conflict of interest.
